# Needs analysis and curriculum development of vocational Chinese for NCS students

**DOI:** 10.1186/2193-1801-3-S1-O3

**Published:** 2014-12-04

**Authors:** Xiao-yan Qiu, Dan-ping Wang, Hau-yee Lo, Ming-tak Tsang

**Affiliations:** Language Centre, Hong Kong Institute of Vocational Education (Shatin), Kragujevac, Hong Kong; Department of General Education, Technical and Higher Education Institute of Hong Kong, Kragujevac, Hong Kong; Language Planning and Development Office, Vocational Training Council, Kragujevac, Hong Kong

## Background

Drawing from the needs analysis approach in curriculum development research, this study focuses on improving the Chinese language learning experience of non-Chinese-speaking (NCS) students in the Vocational Training Council (VTC) through the use of systematic planning and review practices in all aspects of the Chinese language curriculum.

As a cosmopolitan city, Hong Kong has about 340,000 ethnic minority residents, constituting over 5% of the population, according to the 2011 Hong Kong Population Census. Demographic data show that over one half of these NCS people originate from South Asian countries such as Indonesia, the Philippines, India, Pakistan and Nepal. Over the past years, the Hong Kong government has been considering ways to enhance and propagate a harmonious coexistence with these multi-ethnicities. Recent research has found that the NCS minorities have experienced difficulties in integrating into Hong Kong society, particularly in the fields of education and employment. This is largely due to their lack of sufficient Chinese language skills [1]. The current Chinese language curriculum for minority students in schools is not ready to accommodate the learning needs of NCS students. To this end, curriculum-based needs analysis research is urgently needed.

The VTC is dedicated to providing quality Chinese language courses for NCS students. The research team has been carrying out comprehensive curriculum development research and promoting a ‘differentiated teaching’ approach since 2011 [2]. Curriculum development requires needs analysis to devise criteria and a rationale for specific groups of learners through extensive consultation [3]. Furthermore, Richards [4] elaborates that curriculum development is a cyclical process to ‘determine the needs of a group of learners; to develop aims or objectives for a program to address those needs; to determine an appropriate syllabus, course structure, teaching methods, and materials; and to carry out an evaluation of the language program that results from those processes’. In short, this study has two objectives: (1) to identify the learning needs of NCS students and (2) to modify and develop a suitable syllabus and learning materials accordingly.

## Methods

A mixed-method research methodology was adopted to ensure a holistic understanding of the NCS students’ learning needs. Seven secondary schools participated in the research, 328 valid questionnaires were returned and 29 NCS students were interviewed.

## Results

The findings indicate that students’ Chinese proficiency varies, and their general Chinese literacy is insufficient for them to adapt to mainstream classrooms and obtain recognized Chinese proficiency qualifications (e.g. HKDSE), which may result in limited employment opportunities and difficulties in integrating into the Hong Kong community. The interview data show that students have tremendous difficulties in remembering Chinese characters. Furthermore, two literacy tests were conducted by the VTC to examine the development of the students’ basic Chinese reading and writing abilities. Based on the test results from empirical data, the research team moved on to syllabus and materials development to address the identified learning needs, i.e. reading and writing competency.

## Conclusions

Chinese language education in Hong Kong for immigrants and minorities has a profound social-political effect. In this milieu, the VTC has been actively engaging in applied research to provide a refined curriculum to enhance students’ learning experience in Chinese modules. Future research will continue to focus on curriculum and innovative pedagogy development for NCS students to gain better fluency and literacy in Chinese.
